# Stepwise addition of genetic changes correlated with histological change from “well-differentiated” to “sarcomatoid” phenotypes: a case report

**DOI:** 10.1186/s12885-017-3059-1

**Published:** 2017-01-19

**Authors:** Taichiro Goto, Yosuke Hirotsu, Hitoshi Mochizuki, Takahiro Nakagomi, Toshio Oyama, Kenji Amemiya, Masao Omata

**Affiliations:** 1 Department of General Thoracic Surgery, Yamanashi Central Hospital, Yamanashi, Japan; 2 Genome Analysis Center, Yamanashi Central Hospital, Yamanashi, 400-8506 Japan; 3 Department of Pathology, Yamanashi Central Hospital, Yamanashi, Japan

**Keywords:** Sarcomatoid carcinoma, Next generation sequencing, Tumor evolution, *TP53*, *KRAS*

## Abstract

**Background:**

Sarcomatoid cancer is defined by the World Health Organization as a category of non-small cell lung cancers with sarcoma or sarcoma-like differentiation. They are characterized by poor prognosis and resistance to conventional chemotherapy. However, the mutational profile of sarcomatoid cancer remains yet to be elucidated. Sarcomatoid cancers are usually biphasic tumors composed of carcinomatous and sarcomatous components, but the evolutional development of sarcomatoid cancer is controversial.

**Case presentation:**

We present an illustrative case of sarcomatoid cancer composed of three different histological areas. Targeted sequencing of 53 lung cancer-related genes was performed in each component and their phenotypic changes were correlated with stepwise addition of genetic changes.

**Conclusion:**

Sarcomatous change of carcinoma occurs in the case of sarcomatoid cancer, and phenotypic changes to sarcomatoid cancer are associated with the addition of mutation patterns and derived from poorly differentiation tumor.

**Electronic supplementary material:**

The online version of this article (doi:10.1186/s12885-017-3059-1) contains supplementary material, which is available to authorized users.

## Background

Sarcomatoid carcers are rare malignant biphasic tumors composed of carcinomatous and sarcomatous components. However, it is still unclear whether the carcinomatous or sarcomatous component develops first, or whether they develop simultaneously from adjacent areas. We recently encountered a patient who underwent surgery for sarcomatoid cancer that was composed of three different histological areas. To examine whether the different histological components resulted from genetic divergence, targeted sequencing was performed in each histological component. Herein, we report the case findings and the genomic analysis of sarcomatoid differentiation.

## Case presentation

**Fig. 1:**
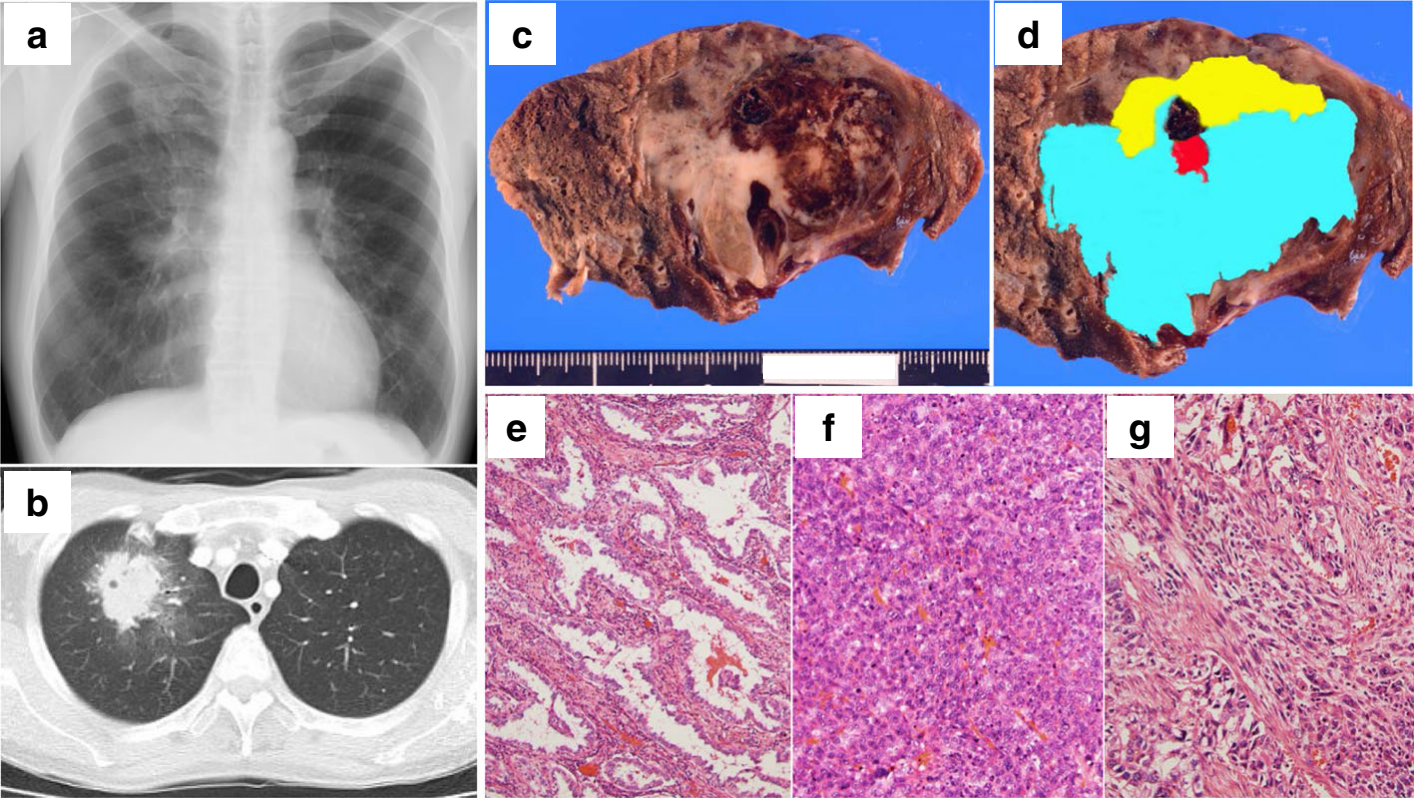
Radiological and histopathological findings. **a** A chest radiograph showing a mass in the right upper lung field. **b** A chest computed tomography scan showing a mass with an irregular surface in the right upper lobe. **c** Gross examination showing a solid tumor (diameter, 5 cm) with internal bleeding. **d-g** Topographic presentation of the cut surface shows three different components; portion A: well-differentiated adenocarcinoma (**e**), portion B: poorly differentiated adenocarcinoma (**f**), and portion C: sarcomatoid cancer (**g**). The ratio of the area occupied by these 3 components, i.e. well-differentiated adenocarcinoma: poorly differentiated adenocarcinoma: sarcomatoid cancer, is 15: 80: 5. *Yellow*, *light blue* and *red* denote the area of well-differentiated adenocarcinoma, poorly differentiated adenocarcinoma, and sarcomatoid cancer, respectively

The patient was a 54-year-old man. He complained of hemosputum, and chest X-ray showed a mass shadow in the right upper lung field (Fig. 1a). He had a history of smoking 20 cigarettes per day for 30 years. Chest computed tomography revealed an irregularly-shaped mass in segment 1 of the right lung (Fig. 1b). Bronchoscopic biopsy led to a diagnosis of adenocarcinoma.

**Fig. 2:**
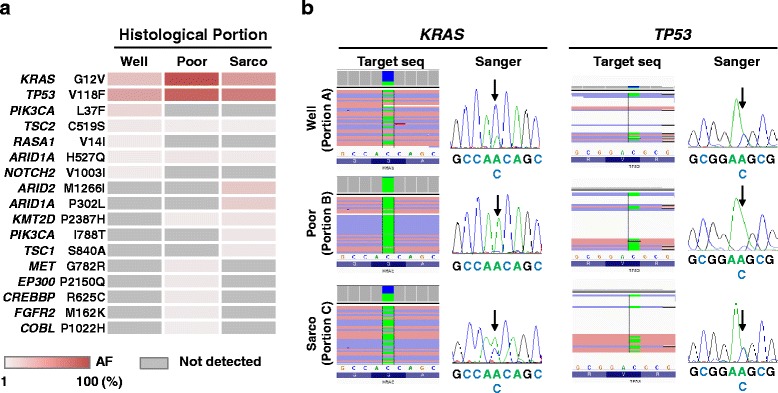
Genomic analyses. **a** Heat map for the mutations in each cancer component and ctDNA detected in plasma. Column next to heatmap shows the mutated genes and amino acid changes. *AF*, allele fraction **b** Validation of somatic mutations by Sanger sequencing. Representative image of read alignments visualized with IGV of targeted sequencing data (*left*) and Sanger sequencing data (*right*). The arrow shows the position of the variant. Well, well-differentiated adenocarcinoma; Poor, poorly differentiated adenocarcinoma; Sarco, sarcomatoid carcinoma

Under a diagnosis of lung cancer (cT2aN0M0), he underwent right upper lobectomy and lymph node dissection. Bilateral legs had been swelling preoperatively, which subsided spontaneously after the surgery. Pleural lavage cytology led to a diagnosis of disseminated adenocarcinoma (pT2aN0M1a, stage IV); histopathological analysis showed that tumor was composed of three different histological areas; portion A: well-differentiated adenocarcinoma, portion B: poorly differentiated adenocarcinoma, portion C: sarcomatoid cancer, and portion B was a major component (Fig. 1c-g). As the sarcomatoid cancer component accounted for less than 10% of the entire tumor area, the cancer did not meet the diagnostic criteria for pleomorphic carcinoma. To examine whether different histological components resulted from genetic divergence, tumor cells of each different histological area were collected from formalin-fixed paraffin-embedded tissues by using laser capture microdissection (Fig. 1d-g). A panel targeting the exon of 53 lung cancer-associated genes was established *in-house*, as we previously reported [1–4]. Subsequently targeted sequencing of those 53 lung cancer-related genes was performed. The sequencing data were processed using standard Ion Torrent Suite software running on a Torrent Server (Thermo Fisher Scientific). Variants calling were performed using an Ion Reporter Server System (Thermo Fisher Scientific), and peripheral blood DNA was used as a control to detect somatic mutations in tumours using filtration of “confident somatic variants” in a Tumour-Normal pair pipeline [4, 5]. This study was approved by the institutional review board, and the patient provided written informed consent. In total, there were 26 mutations found in the cancer (Fig. 2a and Additional file 1: Table S1), and mutations with an allele fraction ≥30% were determined as significant mutations (Table 1). Common missense mutations in *TP53* and *KRAS* genes were detected in the portion A, B and C (Table 1, Fig. 2a). These mutations were validated by Sanger sequencing (Fig. 2b). The positions and patterns of the amino acid changes and base-pair substitutions in those co-mutations were consistent (Table 1).

**Table 1:** Mutation analysis in different histological areas

Histological area	Gene	Mutation	Position	Ref	Variant in tumor	AF
Portion A: well diff. Adeno	*TP53*	p.V118F	chr17:7578461	C	A	47%
	*KRAS*	p.G12V	chr12:25398284	C	A	30%
Portion B: poorly diff. Adeno	*KRAS*	p.G12V	chr12:25398284	C	A	97%
	*TP53*	p.V118F	chr17:7578461	C	A	86%
Portion C: sarcomatoid	*TP53*	p.V118F	chr17:7578461	C	A	70%
	*KRAS*	p.G12V	chr12:25398284	C	A	52%

The table listed somatic mutations with an allele fraction ≥30%

*chr chromosome,* *Ref* reference sequence, *AF* allele fraction

**Fig. 3:**
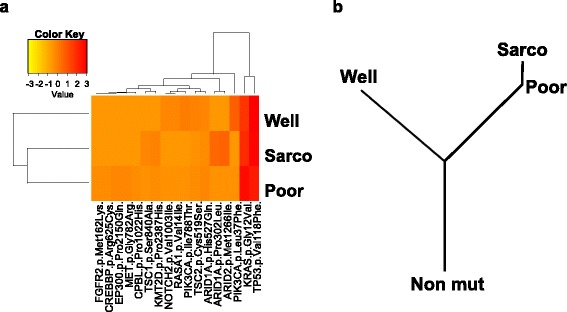
Phylogenetic analyses. **a** Cluster analysis of the point mutations in each cancer component. The mutation data were standardized and presented as a heat map. Column next to heatmap shows the mutated genes and amino acid changes. **b** The phylogenetic tree was constructed by an approach using the bootstrap. Branch lengths correlates with the number of nucleotide substitutions. Abbreviations: Non mut, non-mutated cells; Well, well-differentiated adenocarcinoma; Poor, poorly differentiated adenocarcinoma; Sarco, sarcomatoid carcinoma

For further inference of phylogenies and estimation of evolutionary distances, the Neighbor-Joining method was implemented to cluster the nonsilent mutations and the phylogenetic tree was constructed (Fig. 3a and b) [6]. The ‘ape’ and ‘phangon’R (3.2.3 on linux) packages were utilized for these analyses. As a result, sarcomatoid component clusters together with poorly differentiated adenocarcinoma and that cluster segregates away from well-differentiated adenocarcinoma (Fig. 3a). From the phylogenetic tree, it can be inferred, under the assumption of normal cells without any mutations being roots, that well-differentiated adenocarcinoma first branched off from the roots, and that another branch evolved through poorly differentiated adenocarcinoma into sarcomatoid carcinoma (Fig. 3b). According to the branch length, which indicates the evolutionary distance, the distance between the poorly differentiated adenocarcinoma and the sarcomatoid carcinoma is relatively short.

Postoperative adjuvant chemotherapy was performed, but the patient developed lung and brain metastases and died of cancer 5 months postoperatively.

## Discussion

Sarcomatoid carcinomas are carcinomas with sarcoma or sarcoma-like features. They are rare entities and are characterized by poor prognosis and resistance to conventional chemotherapy or radiotherapy. On evaluating the mutational profile of sarcomatoid cancer, Fallet et al. reported that the most frequently detected mutations were *KRAS*, *EGFR*, *TP53*, *STK11*, *NOTCH1*, *NRAS*, and *PIK3CA* [7].

In our case, comparative genetic analysis of different histological areas revealed intratumoral homogeneity for some mutations. These concordant mutations across different sites reinforced the hypothesis that a single cell clone evolved and progressed later by addition of other mutations into different histological phenotypes, involving epithelial-to-mesenchymal transition. In portions B and C of the tumor, *TP53* and *KRAS* mutations were found at high allelic fractions over 70%, which strongly indicates the incidence of allelic losses or amplifications of these genes.

Some hypotheses have been proposed regarding the development of sarcomatoid carcinoma, such as: (i) simultaneous malignant transformation of epithelial elements and stroma, (ii) malignant transformation of cancer-derived stroma, (iii) sarcomatous change of carcinoma, (iv) carcinomatous change of sarcoma. With regard to the development of sarcomatoid carcinoma in the present case, the mutation analysis results indicated the occurrence of a sarcomatous change.

From the phylogenetic analyses, it can be concluded that stepwise addition of genetic changes caused the sarcomatous change in this cancer. Sarcomatous cancer is reported to be resistant to conventional chemotherapy or radiation therapy and to show poor prognosis. Reflecting on these observations, the mutations responsible for those phenotypic changes may be relate to worse clinical outcomes. Further understanding of the molecular pathology of sarcomatoid cancer may help in selection of sarcomatoid cancer patients for newly developed molecular-targeted therapies.

## Conclusions

The different histological components in lung cancer result from genetic divergence. Sarcomatous change of carcinoma occurs in the case of sarcomatoid or pleomorphic cancer. Phenotypic changes to sarcomatoid cancer are associated with the addition of mutation patterns.

## Additional file

Additional file 1:
**Table S1.**Somatic mutations identified by targeted sequencing (XLSX 13 kb)

## Abbreviations

*EGFR*: Epidermal growth factor receptor
*KRAS*: Kirsten rat sarcoma viral oncogene homolog
*NRAS*: Neuroblastoma ras oncogene
*PIK3CA*: Phosphatidylinositol 3-kinase catalytic, alpha polypeptide
*STK11*: Serine/threonine kinase 11
*TP53*: Tumour protein p53
